# Long Island College Hospital

**Published:** 1868-07

**Authors:** 


LONG ISLAND COLLEGE HOSPITAL.—Professors Austin Flint, Senior and Junior, and Foster Swift, have resigned their respective chairs in this institution.

